# A retrospective study of sugammadex for reversal of neuromuscular blockade induced by rocuronium in critically ill patients in the ICU

**DOI:** 10.1038/s41598-022-04818-7

**Published:** 2022-01-18

**Authors:** Răzvan Bologheanu, Paul Lichtenegger, Mathias Maleczek, Daniel Laxar, Eva Schaden, Oliver Kimberger

**Affiliations:** 1 Department of Anaesthesiology and General Intensive Care, Medical University of Vienna, Vienna, Austria; 2 Ludwig Boltzmann Institute for Digital Health and Patient Safety, Vienna, Austria

**Keywords:** Health care, Medical research

## Abstract

Sugammadex has been approved for reversal of neuromuscular blockade by vecuronium and rocuronium in adults undergoing surgery. Although widely used in the operating room, sugammadex has not been investigated in the intensive care unit setting. This study aimed to evaluate the use of sugammadex in critically ill patients with a focus on known drug-related adverse reactions. In this single-center, retrospective, observational study, 91 critically ill patients who were administered sugammadex while in the ICU were evaluated. Electronic health records were reviewed, and baseline data, as well as indication and incidence of complications possibly related to sugammadex, were retrospectively collected. The most common procedures requiring neuromuscular blockade followed by reversal with sugammadex were bronchoscopy, percutaneous dilatative tracheostomy, and percutaneous endoscopic gastrostomy. Within 2 h following administration of sugammadex, skin rash and use of antihistamines were reported in 4 patients (4.4%) in total; bradycardia was observed in 9 patients (9.9%), and respiratory adverse events were described in 3 patients (3.3%). New-onset bleeding up to 24 h after sugammadex was reported in 7 patients (7.7%), 3of whom received transfusions of packed red blood cells. Sugammadex was well tolerated in critically ill patients and could be considered for reversal of neuromuscular blockade in this population. Larger prospective studies are required to determine the safety profile and evaluate the potential benefit and indications of sugammadex in the critical care setting.

## Background

Sugammadex is a selective antagonist of aminosteroid neuromuscular blocking agents (NMBA) vecuronium and rocuronium approved by the FDA for reversal of neuromuscular blockade in adults undergoing surgery^1^. Sugammadex works by encapsulating the molecules of the NMBA resulting in water-soluble complexes in plasma, which favors dissociation from their targets, the post-synaptic acetylcholine receptors, located at the myoneural junction^2^. Its unique mechanism of action is responsible for the rapid and efficient reversal of the deep neuromuscular blockade, without autonomic cholinergic side effects^3^.

Despite its rising popularity, the routine use of sugammadex has been a subject of debate. The higher price compared to the alternative antagonists of NMBA has limited its use and safety concerns have been raised. The reportedly high incidence of hypersensitivity reactions and anaphylaxis has led to a delayed approval in the US compared to Europe^4^. Other significant adverse reactions, such as cardiac arrhythmia, interference with the coagulation system and increased risk of bleeding, residual neuromuscular block, and bronchospasm have also been reported^5^. Sugammadex is eliminated mainly by renal excretion and is not recommended in patients with a glomerular filtration rate (GFR) < 30 ml/min, even though the clinical significance of its reduced plasma clearance in patients with renal failure is unclear and sugammadex appears to be safe in this population^6,7^.

NMBA are frequently used in the intensive care unit (ICU) to facilitate endotracheal intubation and other bedside procedures performed in general anesthesia, such as bronchoscopy, gastrointestinal endoscopy, and percutaneous dilatative tracheostomy^8^. Post-procedural extubation or return to spontaneous breathing require spontaneous recovery or pharmacologic reversal of neuromuscular blockade. Sugammadex has not yet been investigated in ICU setting and data regarding the safety of sugammadex in critically ill patients is lacking. Due to organ dysfunction and decreased physiological reserve, ICU patients have a higher risk for and are more likely to suffer harm from adverse drug reactions^9^. We performed, therefore, a retrospective review of an electronic health records database to evaluate the use patterns and drug-related adverse reactions associated with sugammadex in the ICU.

## Methods

### Study design and patients

The present study was a retrospective single-center study conducted at the Vienna General Hospital, a university hospital with 60 ICU beds managed by the anesthesiology department and approximately 2500 ICU admissions per year. The Strengthening the Reporting of Observational Studies in Epidemiology (STROBE) guidelines were followed^10^. All methods were performed in accordance with local regulations and the ethical approval (EK Nr. 2273/2020, granted on 18 December 2020 by the Ethics Committee of the Medical University of Vienna, Vienna, Austria). We included all critically ill patients who were administered sugammadex while in the ICU except patients who received sugammadex postoperatively on the first day of the ICU stay, and pediatric patients.

### Data sources

For the current study, data was queried from a reporting database comprising electronic health records of all patients admitted to the ICU since September 1, 2013. Demographics, morphometrics, and clinical data were collected retrospectively. Baseline characteristics, including admission diagnosis, comorbidities, kidney function and use of renal replacement therapy, as well as procedures requiring neuromuscular blockade followed by reversal with sugammadex, dose of NMBA and sugammadex, airway management and ventilation mode, hemoglobin concentration, coagulation tests, and the incidence of known adverse drug reactions were recorded.

### Adverse drug reactions

The incidence of adverse drug reactions was determined using clinical data from the electronic ICU charts. Based on the safety profile of sugammadex in adults undergoing surgery, we investigated the occurrence of possible, previously reported, adverse reactions to sugammadex^11^. We used notes by ICU team members and physiological parameters, laboratory tests, including hemoglobin concentration and coagulation tests, and non-routine orders of corticosteroids, adrenalin, bronchodilators, as well as antiarrhythmic, anticholinergic, and antihistaminic drugs. Heart rhythm disturbances, including bradycardia, asystole, atrial fibrillation, and malignant arrhythmias occurring within 2 h after administration of sugammadex were determined from the recorded heart rate values and the heart rhythm documented by the ICU team. We also screened the ICU charts for electrical defibrillation, cardio-pulmonary resuscitation, and antiarrhythmic and anticholinergic medication. Similarly, drug hypersensitivity reactions were defined either based on the clinical features recorded by the staff, such as skin eruption, bronchoconstriction, hypotension, angioedema, or anaphylactic reactions, or use of antihistaminic drugs. We considered new-onset bleeding any relevant blood loss that became apparent only after sugammadex was administered as documented by the ICU team, regardless of the source of bleeding. To identify occult hemorrhage, hemoglobin concentration values before and after sugammadex use were collected. Respiratory adverse events were considered deterioration of the ventilation or oxygenation compared to baseline, failure to extubate or failed weaning to spontaneous breathing, and bronchospasm on clinical examination by the staff.

### Statistical analysis

Continuous variables are presented as median with interquartile range (IQR) or mean with standard deviation, depending on their distribution. Categorical data are reported as absolute number and relative frequency. To detect episodes of occult bleeding, the variation of hemoglobin concentration relative to sugammadex administration was analysed by fitting a fixed effects model on the hemoglobin concentration values measured in patients without overt bleeding or transfusion of packed red blood cells with time dummy variables and the procedure performed prior sugammadex as regressors. Statistical analysis was performed using the *R* software (R Foundation for Statistical Computing, Vienna, Austria) and the *python* scientific environment^12,13^.

### Ethics approval and consent to participate

Ethics approval, with the reference number EK Nr. 2273/2020, was granted on 18 December 2020 by the Ethics Committee of the Medical University of Vienna, Vienna, Austria, which waived the requirement to obtain informed consent.

## Results

### Patient population

**Figure 1:**
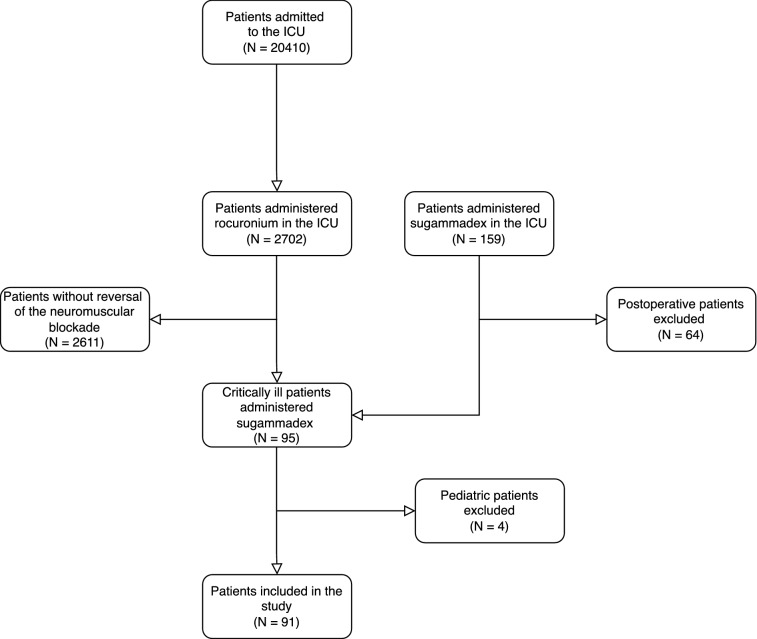
Summary of patient flow diagram. Diagram created using draw.io (Version 15.3.0), JGraph Ltd, Northampton, UK. URL: https://diagrams.net/.

159 critically ill patients admitted to the ICU after September 1, 2013 received sugammadex while being treated in the ICU. After excluding 4 pediatric patients and 64 patients who received sugammadex postoperatively within 24 h following their ICU admission, 91 patients were included in the analysis (Fig. 1).

### Patients’ characteristics

Demographics, morphometrics, and diagnostic data are presented in Table 1. The most common bedside procedures performed were bronchoscopy, percutaneous dilatational tracheostomy, and percutaneous endoscopic gastrostomy (Table 2). The median dose of rocuronium bromide was 0.7 mg kg^−1^ (IQR 0.5–1.1 mg kg^−1^). The usual dose for rapid sequence induction of 0.9–1.2 mg kg^−1^ was exceeded in 10 patients. The median dose of sugammadex was 2.5 mg kg^−1^ (IQR 2.2–2.9 mg kg^−1^) actual body weight and no obvious dose adjustments were observed in obese patients. The depth of the neuromuscular block could not be reviewed, as it was either not used or not documented in the electronic ICU chart.

**Table 1:** Patients’ main characteristics.

Variables	–
Male n (%)	61 (67%)
Age median (IQR)	63 (52–73)
Weight median (IQR)	78 (70–88)
BMI median (IQR)	25.9 (23.2–28)
Obesity^a^ n (%)	10 (10.9%)
**Reason for admission**^**b**^	–
medical n (%)	17 (18.7%)
surgical n (%)	74 (81.3%)
Planned admission n (%)	73 (80.2%)
**Surgical diagnosis**	–
General surgery n (%)	19 (25.7%)
Trauma surgery n (%)	14 (18.9%)
Thoracic surgery n (%)	11 (14.9%)
Transplant surgery n (%)	7 (9.5%)
Heart surgery n (%)	7 (9.5%)
Neurosurgery n (%)	6 (8.1%)
Other n (%)	10 (13.5%)
**Medical diagnosis n (%)**	–
Respiratory	8 (47.1%)
Cardiovascular	5 (29.4%)
Neurologic	2 (11.8%)
Digestive	1 (5.9%)
Metabolic	1 (5.9%)
**Comorbidities n (%)**	–
COPD^c^	19 (20.8%)
Asthma	2 (2.2%)
Chronic kidney disease	11 (12%)
Chronic liver disease	4 (4.4%)
Heart failure	22 (24.1%)
Atrial fibrillation	20 (21.9%)
**Renal function**	–
Patients on enal replacement therapy n (%)	21 (23%)
Patients successfully weaned from renal replacement therapy n (%)	13 (14.3%)
Creatinine clearance < 30 mL/min	9 (9.9%)

*IQR* interquartile range, *BMI* body mass index.

^a^BMI > 30 kg/m^2^.

^b^from the primary diagnosis for the ICU admission.

^c^Chronic obstructive pulmonary disease.

**Table 2:** Procedures requiring neuromuscular blockade followed by administration of sugammadex.

Procedure	n (%)	Duration in minutes median (IQR)	Day of ICU stay median (IQR)
Bronchoscopy	27 (29.7%)	27 (18–60)	7 (4–17)
Percutaneous dilatative tracheostomy	25 (27.5%)	45 (35–60)	8 (6–14)
Percutaneous endoscopic gastrostomy	13 (14.3%)	60 (48–60)	23 (17–28)
Gastrointestinal endoscopy	11 (12%)	61 (44–91)	8 (5–26)
Surgical wound management	7 (7.7%)	60 (53–80)	10 (6–15)
Other	8 (8.8%)	52 (32–78)	3 (2–17)

*IQR* interquartile range.

### Adverse drug reactions

Sugammadex was used to allow extubation in patients who, before the procedure, were breathing spontaneously without an airway device. In patients who underwent assisted ventilation before the procedure, respiratory weaning and returning to spontaneous breathing after the procedure were attempted. 43 patients (47.2%) were successfully extubated after a median time of 15 min following administration of sugammadex (IQR 6–40 min). 43 patients (47.2%) still requiring ventilatory assistance post-procedurally were weaned to spontaneous or assisted breathing after a median time of 30 min (IQR 15–70 min). One patient (1.1%) was reintubated, while 4 patients (4.4%) could not be weaned and remained in controlled ventilation mode despite having received sugammadex. No severe respiratory events were observed; however, bronchospasm was documented in 3 patients (3.3%) and supplementary, non-routine bronchodilators were used up to 2 h following administration of sugammadex in 6 patients (6.6%).

One patient presented with skin rash and 3 other patients were administered antihistamines. Allergen testing was not performed in any of these cases and previous exposures to sugammadex were documented. Supplementary corticosteroids were administered in 4 patients (4.4%) after sugammadex, in combination with either antihistamine medication (in 2 patients) or bronchodilators (in 1 patient).

New-onset bleeding episodes documented within 24 h after sugammadex occurred in 7 patients (7.7%) and 3 patients required transfusion of packed red blood cells. New-onset bleeding occurred from the tracheostomy site, surgical site, and respiratory tract following bronchoscopy and transbronchial lung biopsy and was in every case related to the procedure performed. Global coagulation tests did not reveal any significant abnormalities, nor did these patients receive anticoagulants in the therapeutic dose range. 13 patients who had already presented with hemorrhage before sugammadex was administered, of whom one patient had been prescribed higher than prophylactic doses of low-molecular-weight heparin, and 2 patients were administered an antiplatelet drug, were not included in the group of patients with new-onset bleeding. In patients without documented bleeding episodes or transfusion of packed red blood cells, the fixed effects model fitted on the measured values of hemoglobin concentration did not reveal a significant drop in hemoglobin levels after sugammadex.

No cases of malignant arrhythmias, asystole, or cardiac arrest occurred. 9 patients (9.9%) developed sinus bradycardia, which was self-limited or required no intervention in 7 patients, while 2 others were administered anticholinergic medication. 2 of the patients had similar episodes of bradycardia before administration of sugammadex and 4 patients were being treated with betablockers. Of note, 8 patients (8.8%) had a permanent or temporary pacemaker while in the ICU.

An overview of the patients where an adverse reaction following administration of sugammadex was documented is presented in Table 3.

**Table 3:** Overview of the patients who experienced adverse events after sugammadex.

Case	Gender	Age	Admission diagnosis category	Procedure	Adverse events	Therapy	Dose of rocuronium bromide (mg kg^−1^)	Dose of sugammadex (mg kg^−1^)	Duration of the procedure (minutes)	Pulmonary comorbidities	Cardiac comorbidities	Concomitant antiarrhythmic therapy	Anticoagulant dose in the ICU	Antiplatelet agent
1	Male	58	Thoracic surgery	Bronchoscopy	Bronchospasm	Bronchodilator therapy	0.38	1.25	105	COPD	HTN	–	Prophylactic	None
2	Male	75	Thoracic surgery	Bronchoscopy	Failure to wean	Controlled ventilation	0.54	2.17	30	–	AFib CAD	Beta-Blocker	Therapeutic	None
3	Male	67	Visceral surgery	Bronchoscopy	Bradycardia		1.13	2.82	61	–	HTN AFib	Beta-Blocker	Therapeutic	None
4	Male	75	Thoracic surgery	Bronchoscopy	Bradycardia		0.42	4.21	60	–	De novo AFib	Beta-Blocker	Therapeutic	None
5	Male	74	Medical Respiratory	Bronchoscopy	Bradycardia	Anticholinergic drug	0.43	2.17	15	–	AFib	Beta-Blocker	Therapeutic	None
6	Male	76	Visceral surgery	Bronchoscopy	Bronchospasm New-onset pulmonary bleeding Reintubation	Bronchodilator therapy Controlled ventilation	1.05	2.11	230	COPD	HTN AFib	Digitoxin	Prophylactic	None
7	Male	55	Medical respiratory	Bronchoscopy	New-onset pulmonary bleeding	Packed RBC transfusion	0.59	2.35	16	–	HTN AFib	Amiodarone	Prophylactic	None
8	Female	84	Neurosurgical	External ventricular drain	Bradycardia	-	0.71	2.86	78	–	HTN AFib CAD	–	No anticoagulation	None
9	Male	64	Medical cardiovascular	Percutaneous endoscopic gastrostomy	Failure to wean	Controlled ventilation	0.91	1.82	90	–	AFib HFrEF	Beta-Blocker	Prophylactic	None
10	Female	52	Visceral surgery	Surgical wound management	New-onset bleeding from surgical site	Packed RBC transfusion	0.54	2.17	60	–	–	–	Prophylactic	None
11	Female	64	Vascular surgery	Surgical wound management	Bradycardia	Anticholinergic drug	0.91	3.64	60	COPD	HTN	Beta-Blocker	Prophylactic	None
12	Female	32	Trauma surgery	Surgical wound management	New-onset bleeding from surgical site	Packed RBC transfusion	0.88	3.51	90	–	–	–	Prophylactic	None
13	Female	96	Visceral surgery	Surgical wound management	Skin rash	–	1.82	4.55	47	–	–	–	Prophylactic	None
14	Female	87	Cardiac surgery	Percutaneous tracheostomy	New-onset bleeding from tracheostomy site Failure to wean	–	1	4	60	–	HTN CAD	Beta-Blocker	Prophylactic	None
15	Male	64	Medical cardiovascular	Percutaneous tracheostomy	Bronchospasm New-onset bleeding from tracheostomy site	Bronchodilator therapy	0.63	2.50	60	COPD	HTN CAD HFrEF	Beta-Blocker	Prophylactic	Aspirin
13	Female	59	Neurosurgery	Percutaneous tracheostomy	Bradycardia	–	1.33	2.67	58	–	De novo AFib	Amiodarone	No anticoagulation	None
14	Male	50	Thoracic surgery	Percutaneous tracheostomy	New-onset bleeding from tracheostomy site	Packed RBC transfusion	0.75	5.97	10	COPD	–	–	No anticoagulation	None
15	Male	66	Trauma surgery	Percutaneous tracheostomy	Bradycardia	–	1.11	2.22	30	–	HTN HFrEF	Beta-Blocker	Prophylactic	None
16	Female	61	Vascular surgery	Percutaneous tracheostomy	Failure to wean	–	1.33	2.67	45	–	De novo AFib	Amiodarone	No anticoagulation	None
17	Male	63	Medical respiratory	Percutaneous tracheostomy	Bradycardia	–	1.43	2.86	90	–	HTN CAD	Beta-Blocker	Prophylactic	Aspirin
18	Female	23	Trauma surgery	Percutaneous tracheostomy	Bradycardia	–	0.64	2.56	30	–	–	Beta-Blocker	Prophylactic	None

*COPD* chronic obstructive pulmonary disease, *HTN* arterial hypertension, *AFib* atrial fibrillation, *CAD* coronary artery disease, *RBC* red blood cells, *HFrEF* heart failure with reduced ejection fraction.

## Discussions

Sugammadex was generally well tolerated in critically ill patients undergoing short bedside procedures performed by intensivists requiring neuromuscular blockade. Sugammadex has been approved by the FDA in adults undergoing surgery and data on its efficacy and safety in the intensive care setting is missing, although anecdotally, it is being increasingly used in the ICU in our center. While “off-label” medication use itself is not associated more frequently with adverse drug reactions in the ICU^14^, drug-related adverse reactions and events are generally more frequent and may cause more harm due to the complexity of the ICU environment and the labile state of the critically ill^9^. In the present study, however, the adverse reactions observed after sugammadex use in ICU patients were not severe.

The potential of hypersensitivity reactions to sugammadex, including anaphylactic reactions, has been widely reported and remains the primary safety concern, despite its low incidence in surgical patients and healthy volunteers^15,16^. We identified one case of skin rash and 3 other patients who received antihistamines up to 2 h after administration of sugammadex, yet none of the patients experienced concomitant respiratory symptoms or hemodynamic instability. Notably, intradermal allergen testing was not performed in any of the cases and the causative agent could not be ultimately identified, although specific workup is required after suspected drug hypersensitivity reactions, to allow accurate diagnosis and appropriate preventive measures.

New-onset bleeding from tracheostomy site, surgical site, or respiratory tract was reported after sugammadex in 7 patients. However, the anticoagulant effect of sugammadex remains controversial. Initial data on healthy volunteers showed a transient and minor prolongation of the activated partial thromboplastin time (aPTT)^11^. The effect on coagulation parameters has been since attributed to an in vitro artefact due to a lipid binding effect of sugammadex without clinical relevance^17^. Furthermore, hemorrhage is a common post-procedural complication in the ICU, with as many as half of patients suffering from mild bleeding following percutaneous tracheotomy^18^.

Cardiac arrhythmia, specifically bradycardia and asystole, have been reported with sugammadex, particularly in patients with underlying cardiac disease^19^. Bradycardia occurred in 9.9% critically ill patients after administration of sugammadex and was either self-limited or responded well to anticholinergic medication and was not associated with hemodynamic instability.

Weaning failure occurred rarely and was attributed rather to the underlying critical illness than residual neuromuscular blockade or respiratory adverse reaction to sugammadex. However, due to the design of electronic ICU chart, the depth of the neuromuscular block could not be reviewed. Consequently, one of the major limitations of our study, is that no detailed conclusions about the recovery, reversal and residual neuromuscular block can be drawn, and residual neuromuscular blockade cannot be ruled out as cause for weaning failure or respiratory adverse events. Moreover, failure to document the level of neuromuscular blockade and appropriate reversal or recovery in patients where neuromuscular blocking agents have been used is sub-optimal, as such monitoring is considered standard in anesthesia and critical care practice in all patients who are administered neuromuscular blocking agents^8,20^. In our cohort 6 patients were administered supplementary bronchodilator medication and bronchospasm was documented in 3 patients. While sugammadex has also been associated with respiratory complications^21^, in the current study bronchospasm was only documented in patients with obstructive respiratory disease diagnosed prior to the ICU admission who underwent airway instrumentation.

We acknowledge that the small sample size and the retrospective study design may further limit the validity of these findings. While the study is not adequately powered to detect infrequent adverse reactions, electronic health records are being increasingly used for detection of adverse drug reactions^22^. However, inherent limitations of database reviews, due to the limited quality of data through possible charting errors, missed adverse events, and underreporting of adverse events or incomplete documentation cannot be excluded. Furthermore, the causality between sugammadex and the adverse events observed is uncertain. We identified adverse drug reactions that are known to be associated with sugammadex and occurred within a limited timeframe after sugammadex was administered. However, critically ill patients are complex, have multiple comorbidities, and are simultaneously exposed to several drugs and interventions. Overall, the potential risk of side effects that can be attributed to sugammadex remains unknown but is likely to be low. Future research will have to address not only the safety, but also the benefit of sugammadex in the ICU setting. Although sugammadex appears to be safe in critically ill patients in this retrospective case series, its impact on measurable clinical outcomes, such as length of ICU stay, is unknown, considering the small reduction in the duration of mechanical ventilation that can be achieved.

## Conclusions

We describe 91 critically ill patients who were administered sugammadex for reversal of neuromuscular blockade induced by rocuronium following bedside procedures in the ICU. Sugammadex was well tolerated, as no severe adverse reactions were observed. The use of sugammadex in critically ill patients is “off-label” and further research is needed to accurately determine the safety profile and role of sugammadex in this population.

## Data Availability

The datasets analysed during the current study are available from the corresponding author on reasonable request.

## Abbreviations

aPTT: Activated partial thromboplastin time
FDA: Food and Drug Administration
ICU: Intensive care unit
IQR: Interquartile range
NMBA: Neuromuscular blocking agent
